# Patients’ experiences with GLP1-RAs – a systematic review

**DOI:** 10.1080/02813432.2025.2477141

**Published:** 2025-03-12

**Authors:** Christoffer Kraul Ibsen, Marius Brostrøm Kousgaard, Sofie Olsen, Ann-Kathrin Lindahl Christiansen, Catharina Thiel Sandholdt, Rasmus Rørth, Gritt Overbeck

**Affiliations:** a Center for General Practice, University of Copenhagen, Copenhagen, Denmark; b Rigshospitalet Department of Hormonal and Metabolic Diseases, Copenhagen, Denmark

**Keywords:** Lived experiences, GLP1-RA, systematic review, perception, perspective

## Abstract

**Background:**

Obesity is a complex condition and a recognized public health challenge. Previous treatment options were associated with high failure rates, but recent trials have shown that significant weight loss can be achieved with GLP1-RAs. However, little is known about the patient’s experiences with GLP1-RAs.

**Objectives:**

This paper systematically reviews research on patients’ experience with GLP1-RAs.

**Methods:**

A literature search in PubMed, PsycINFO, Embase and Sociological Abstracts included studies on adults’ experiences with GLP1-RAs, regardless of methodology. Exclusions of studies: mental illness, pregnancy, former bariatric surgery, PCOS. Study quality and transparency were assessed according to design, using thematic analysis for synthesis.

**Results:**

Nine studies, selected from 7,607 records, encompassed three qualitative studies (semi-structured interviews), three RCTs, two narrative reviews and one survey study. The analysis identified five key themes: (1) Patients are willing to accept adverse events, like gastrointestinal disorders, for successful weight loss, (2) Patients experience improved physical functioning, well-being, and active daily living as a result of weight loss, (3) Patients express diverse opinions and skills regarding the medication’s usability, (4) Patients believe that the medication improves their ability to manage sweet cravings, (5) Gender seems to affect patients’ experiences with the medication, with females reporting more benefits than males.

**Conclusion:**

Despite a huge demand and usage of GLP1-RAs, qualitative research on patients’ experiences is scarce. Further studies are crucial for understanding short and long-term patient experiences.

## Background

**Figure 1.**
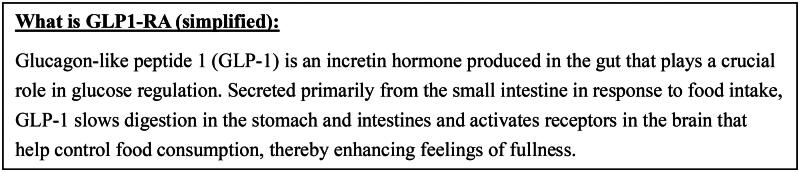
What is GLP1-RA (simplified).

People living with obesity face a myriad of challenges regardless of age and socioeconomic background. Treating obesity has traditionally been difficult and associated with high failure rates, but the glucagon-like peptide 1 receptor agonists (GLP1-RAs), currently seems to be transforming the treatment of obesity [1–4]. The most commonly used GLP1-RAs worldwide are exenatide, liraglutide, dulaglutide, lixisenatide, semaglutide and tirzepatide, see Figure 1 for further details [5]. Especially, trials with semaglutide showed that significant weight loss can be achieved with the new weight loss medication [1–4]. This have created a massive increase in the demand for these drugs and simultaneously increased the pressure on the medical professionals prescribing the drugs [6,7].

Semaglutide was originally approved for type 2 diabetes in 2017 under the trade name Ozempic, mainly because of its ability to enhance glucose-dependent insulin secretion, decreasing glucose levels in patients with diabetes [6,8]. Not only did semaglutide lower glucose levels, but patients also experienced weight loss as a secondary outcome of consuming Ozempic [9].

The newest semaglutide, Wegovy, can be administered in higher subcutaneous doses up to 2.4 mg and can, currently only be administered through weekly subcutaneous self-injections [8]. The Indication for prescribing Wegovy in Denmark is BMI (kg/m^2^) ≥ 30 or ≥ 27 to < 30 with at least one weight-related coexisting condition (hypertension, diabetes, dyslipidemia, obstructive sleep apnea or cardiovascular disease). Pharmacological weight loss treatment like Wegovy ought to always be initiated during continued lifestyle interventions [10].

When new health technologies are implemented, both (1) clinical effects, (2) financial costs, (3) organizational consequences and (4) patient perspectives should optimally be evaluated. The clinical effects of semaglutide on weight and HbA1c reduction are well established in the SUSTAIN (2017), PIONEER (2019) and STEP studies (2021)[1–4]. Also, the financial cost of semaglutide has been analyzed [11]. However, the organizational consequences (cf. the patient pressure on the GPs) and especially the patient perspective have not been sufficiently addressed.

### Patients’ experiences with medication are measured using patient-reported outcomes (PRO)

This involves patients sharing their personal views on how the medication affects their symptoms, daily activities and overall well-being [12].

The STEP studies, which were double-blind randomized control trials, mainly focused on weight loss. However, they also gathered secondary data on patient-reported outcomes. This data was collected through surveys that assessed quality of life, physical function and mental well-being. The results from these surveys were then converted into norm-based scores, where higher scores indicated better quality of life [1,12].

While the STEP studies included surveys to capture aspects of the patients’ experiences, qualitative methods such as interviews can delve into greater detail about patients’ experiences and feelings, than questionnaires [13]. This paper aims to review all available research literature on the patients’ experiences with GLP1-RAs.

## Methods

In reporting this systematic review we have used ENTREQ criteria for enhancing transparency in reporting [14].

### Search strategy

A comprehensive search strategy was created by CKI and GO. We used the Population Exposure Outcome (PEO) format to categorize the search: P: patients, E: GLP1-RAs, O: patients’ experiences with GLP1-RAs, hereby also using relevant synonyms and trade names for the abovementioned categories [15]. Afterward, a search string was developed for PubMed and adjusted to fit PsycINFO, EMBASE and Sociological abstracts (Appendix 1). The searches were performed between 12 August and 12 November 2023, and updated 28 August 2024. Additionally, an exploratory search in Google Scholar alongside with comprehensive database searches using relevant tradenames combined with experience including relevant synonyms (se Appendix 1 for exploratory searches). A backward search was also conducted in PubMed and Google Scholar, looking through relevant citations in articles leading to a wider exploration of the literature [16].

### Study selection

CKI screened the results from the abovementioned databases using the search string; GLP1-RA, combined with experience, attitude, perception, perspective and preference. All searches from the databases were then imported to ENDNOTE [17], where duplicates were electronically discarded. CKI screened the titles and abstracts of the studies in ENDNOTE and discarded studies that did not meet the aim of the systematic review. Approved studies were then imported to COVIDENCE [18], where CKI and GO separately screened titles and abstracts of the imported studies. Sixty-seven articles were discussed face-to-face and studies that did not meet the inclusion criteria were discarded. CKI and GO collaboratively determined and agreed upon the selection of articles for full-text reading.

### Inclusion criteria

Original, peer-reviewed studies available in the above-mentioned databases between 12 August and 12 November 2023, and updated on 28 August 2024, targeting patients’ experiences with GLP1-RA were included, regardless of methodology. Patients had to be at least 18 years old and have experience with GLP1-RA treatment. In this systematic review, an unlimited time frame for the publication of studies was utilized to comprehensively search the available literature as extensively as possible. All patients’ experience of all GLP1-RA products were included for review (see Appendix 1).

### Exclusion criteria

Studies that only included patients under 18 years of age or patients in treatment with antipsychotic medication, patients suffering from suicidal behaviors, patients with former bariatric surgery, poly-cystic ovarian syndrome (PCOS) and pregnant patients were excluded due to coexisting conditions could affect their experience with GLP1-RAs. Non- English studies were also excluded.

### Quality assessment

The quality and transparency of the qualitative included articles were evaluated through the 32-item checklist of Consolidation Criteria for Reporting Qualitative Research (COREQ) [19]. CKI and CTS scored the included articles, individually, any questions that arose were discussed with GO. The Consolidated Standards of Reporting Trials (CONSORT) were used for quality assessment of the RCT studies [20]. Strengthening the Reporting of Observational Studies in Epidemiology (STROBE) was used to assess the quality of the survey study. We did not exclude any studies during the quality assessment phase.

### Data extraction and synthesis of results

The exploratory nature of this systematic review made it relevant to employ an inductive analysis method [21]. We conducted thematic analysis on the findings from all included studies, following the six phases outlined by Braun and Clarke [22]. The analytic approach to the entire data material was inductive, meaning that no predefined framework was applied. We used the following steps: (1) CKI thoroughly familiarized himself with the data by reading and re-reading the extracted results from the included studies, allowing immersion in the content. Initial notes were taken to document early impressions and potential points of interest. (2) Following familiarization, CKI systematically coded the data. This involved identifying and labeling interesting features across the entire data set relevant to patients’ experiences with GLP-RAs. Codes were applied to segments of text that appeared significant, ensuring a consistent approach across all studies included in the review. (3) Once the initial coding was completed, CKI organized the generated codes into potential themes. This phase involved collating codes that shared similarities and grouping them. (4) CKI, in collaboration with GO and MBK, reviewed the initial themes to ensure they captured the essence of the coded data and were consistent across the entire data set. (5) CKI refined the specifics of each theme, ensuring clarity and focus. The themes were defined and named in a way that clearly conveyed their essence and significance to the research question. This involved ongoing analysis to sharpen the definitions and involved all authors. (6) CKI selected vivid extracts that exemplified each theme. These extracts were included in the final report to illustrate the themes and provide a rich, nuanced understanding of patients’ experiences, thoughts, feelings and preferences regarding the use of GLP-RAs.

## Results

**Figure 2.**
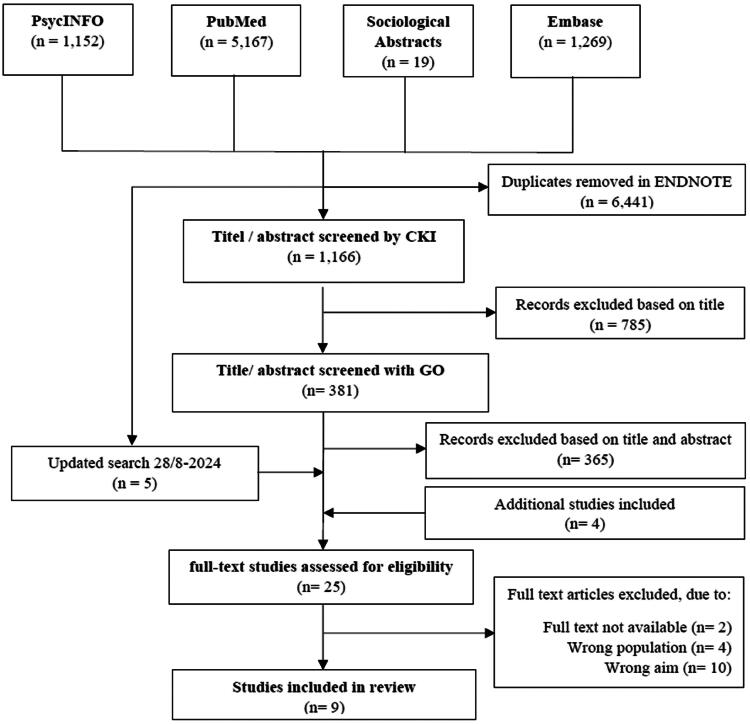
Prisma.

The Prisma figure (Figure 2) illustrates the selection process. The four databases PubMed, Embase, PsycINFO and Sociological Abstracts were searched, and 7,607 references were identified using the search string (Appendix 1), with 6,441 identified as duplicates and removed in ENDNOTE. We excluded 785 studies based on title and abstract, leading to 381 studies being imported into COVIDENCE. When screening articles based on title and abstract, 67 conflicts arose between CKI and GO, which were resolved through discussion. Four additional studies were included, along with an updated search string. Out of 25 studies that underwent full-text reading for eligibility, 4 had the wrong population, 2 lacked full texts and 10 had an inappropriate aim. Ultimately, nine studies were included in the systematic review.

### Study characteristics

Table 1 outlines the characteristics of the nine included studies. Three were qualitative studies using semi-structured interviews to explore how patients with type II diabetes experienced GLP1-RAs [23–25]. The STEP studies, all double-blind randomized control trials (*n* = 3), primarily focused on weight loss but included secondary findings on quality of life, physical functioning, and mental well-being obtained by norm-based surveys which were analyzed with descriptive statistics [1,3,4]. Two studies explored the broader benefits of the STEP studies, specifically focusing on quality of life, mental well-being and physical functioning [26,27]. The final study, an observational prospective survey study, investigated patients’ control of eating in regards to emotional eating, which also had been identified as a theme in the STEP 5 study [28].

**Table 1.** The qualitative studies and narrative reviews.

First author with reference	Year of public-cation	Country	Study objectives	Study description	No. of parti- cipants	Description of participants	Methodology and analysis	Funded by
Holmes-Truscott et al. [24]	2021	Australia	To explore the expectations and experience of adults suffering from type II diabetes and using GLP1-RA.	A qualitative study with face-to-face or telephone semi structured interviews.	19	English-speaking adults living in Victoria (Australia) with type 2 diabetes who had started (but not necessarily continued) GLP-1RA therapy within the past 3 years.	Qualitative interview study	
Matza et al. [23]	2021	GB	To Illustrate patients’ preference and provide an estimation of health state utility in the GLP1-RA therapy process for the treatment of type 2 diabetes, through semi-structured interview.	Observational qualitative study	207	30–75 years old UK resident and diagnosed with type 2 diabetes. Not been treated with semaglutide or dulaglutide before.	Qualitative interview study	Eli Lilly
Matza et al. [25]	2023	USA	To gain insight into the experience of patients receiving Tirzepatide for Type II diabetes.	Cross-sectional qualitative study	28	Adults with type 2 diabetes, inadequately with metformin (≥1500 mg). HbA1c 7,5-10,5% and BMI > 25 and received treatment with Tirzepatide in SURPASS 2.	Qualitative interview study	Eli Lilly
O’Neil et al. [26]	2022	USA	To illustrate and summarize the beneficial effects on quality of life and control of eating in the STEP 1-5 studies for primary care providers.	Narrative review	4,585	Same population as in the STEP 1-5 trials. The STEP 2 study included participants with type II diabetes, and STEP 3 used the same inclusion criteria as STEP 1.	Literature synthesis	Novo Nordisk
Kolotkin et al. [27]	2023	Japan, Sorth Korea	To illustrate the wider benefits of the STEP 6 study, with a specific focus on quality of life and meaningful within-person improvements.	Narrative review	401	≥18 years South Korea ≥20 years Japan BMI: ≥ 27 with two or more treated or untreated weight-related comorbidities. BMI: ≥ 35 with one or more treated or untreated weight-related comorbidities.	Literature synthesis	Novo Nordisk
Wilding et al. [1] (STEP 1)	2021	USA	To show that once-a-week semaglutide can lead to weight loss in a population without type II diabetes.	Doublet blinded randomized control study	1,961	Adults with a self-reported unsuccessful dietary weight loss. >30 BMI or >27 BMI with one weight-related coexisting condition. Participants were from 16 counties in Asia, Europe and South and North America.	Quantitative study	Novo Nordisk
Rubino et al. [3] (STEP 4)	2021	USA	To illustrate the effect of continued once-a-week semaglutide vs. placebo with behavioral interventions in both groups.	Doublet blinded randomized control study	1,051	Same inclusion criteria as in STEP 1. The study was conducted from June 2018 to March 2020. Participants were from DK, USA, Israel, Sweden, Spain, and Switzerland.	Quantitative study	Novo Nordisk
Garvey et al. [4] (STEP 5)	2022	USA	To follow patients in treatment with semaglutide vs. placebo for 2 years. Especially looking at control of eating, quality of life and weight loss.	Doublet blinded randomized control study	304	Same inclusion criteria as in STEP 1. The study was conducted from 5 October 2018 to 1 February 2019. Participants were from Hungary, Spain, Canada, Italy, and USA.	Quantitative study	Novo Nordisk
Nicolau et al. [28]	2022	Spain	The aim of the study was to investigate the effect of semaglutide once weekly on emotional eating, food cravings and psychological well-being among patients with obesity.	Observational prospective study	69	Adults who attended an obesity clinic in Spain from June to September 2021. Who failed to maintain a weight loss despite lifestyle interventions, such as a tailored hypocaloric diet and exercise. >30 BMI or >27 BMI with one weight-related coexisting condition.	Quantitative study	

### Quality assessment

The explicitness and comprehensiveness of the nine included studies were assessed using the COREQ-, CONSORT- and STROBE checklists. Great variability was identified when employing the 32-item COREQ checklist on the qualitative studies, displaying scores ranging from 17 to 28 out of a total of 32, averaging 23.6. The CONSORT checklist was used to evaluate the three included RCT studies with scores ranging from 19 to 21 out of a total of 25, averaging 19.6. It was not possible to retrieve the questionnaires from the RCT, leaving only the SF-36 and IWQOL-Lite-CT norm-based scores. The STROBE checklist was used to assess the single included survey study with a score of 15 out of 22.

### Synthesis of results

Across the nine included studies five major themes were generated in the analysis:

(1) Patients are willing to accept adverse events, like gastrointestinal disorders, for successful weight loss, (data from qualitative interview studies and quantitative studies). (2) Patients experience improved physical functioning, well-being and active daily living as a result of weight loss (data from qualitative interview studies and quantitative studies). (3) Patients express diverse opinions and skills regarding the medication’s usability (data from qualitative interview studies). (4) Patients believe that the medication improves their ability to manage sweet cravings (data from quantitative studies). (5) Gender seems to affect patients’ experiences with the medication, with females reporting more benefits than males (data from qualitative interview studies and quantitative studies).

#### 1. Patients are willing to accept adverse events, like gastrointestinal disorders, for successful weight loss

The most commonly reported negative experiences were gastrointestinal disorders; among these were the most frequently reported nausea, diarrhea, vomiting and constipation [1,3,4,26]. Nausea was the most reported adverse event in the included studies. Nausea was most frequently experienced in the dose escalation period, and thereafter the experience of nausea was reduced [24,25].

‘I would literally switch from frantically starving to within a click of fingers to horrendously nauseous and then back … and this would just go on day and night, day and night, for weeks. So that was kind of horrible and it started to calm down after about three weeks, but gee that was tough…’ (patient with type 2 diabetes, gender and age not given) [24]

Some patients identified nausea and the suppression of appetite as a gastrointestinal signal that the treatment was working, which made them more accepting of feeling nausea:

‘I still get a little bit of nausea, which to me, is really good, because it does put me off eating all the time… As long as the weight comes off…I will put up with anything, as long as it goes.’ (Woman, 28y, with type 2 diabetes) [24]

Vomiting was also frequently reported in the included studies. Like nausea, the vomiting would fade out after 2–3 weeks for most patients. However, for some patients, it did not, but even though one patient had to discontinue her treatment due to severe vomiting and a rash, the patient still wanted to try the same treatment again because of the weight loss.

‘I would like to try it again. I liked the way I felt, because I was losing weight’ (patient with type 2 diabetes, gender and age not given) [25]

For some patients, the motivation and experience of losing weight, therefore, counterbalance the experiences of adverse events.

One in 20 patients reported gallbladder conditions [2]. Gallbladder disorders are well-known adverse events after rapid weight loss, thus unlikely to be directly correlated with semaglutide treatment [1,26]. Few patients experienced severe side effects such as pancreatitis, and even fewer patients worried about thyroid cancer [24].

#### 2. Patients experience improved physical functioning, well-being and active daily living as a result of weight loss

Patients generally reported improvements in their physical functioning when losing weight using semaglutide. The five questionnaire items that assessed physical functioning in the STEP study were 1. Trouble bending over, 2. Tired or winded, 3. Unable to stand comfortably, 4. Not physically active, 5. Unable to walk far/quickly [26]

‘The weight loss has helped me in bending over to pick up things or yard work, my involvement with my grandkids, doing the chores around the house, going down the stairs to do a load of laundry, or just standing. When you’ve lost weight, you can stand at the sink longer. You can do what you got to do longer.’ (interview with man, 64 y with type 2 diabetes) [25]

Some patients improved their walking capacity and gained more motivation for exercise:

‘I noticed the more weight I lost, the more energy I felt. I could walk and walk and not get as winded. I actually felt like wanting to exercise.’ (interview with female, 52 y with type 2 diabetes) [25]

Other patients experienced having more energy and improved active daily living (ADL):

‘I can do more things. And I feel less tired… And standing and sitting and folding up clothes and ironing and putting things away and just keeping the house clean, and then doing my gardening. That was a joy for me.’ (interview with female, 66 y with type 2 diabetes) [25]

Furthermore, the mental component in the SF-36 was improved in the STEP studies. The mental component covers vitality (energy level), social functioning (social activities), emotional role (daily activities impacted by emotional problems) and mental health (happy/sad) [26]. However, patients in the qualitative study also experienced improved happiness:

‘After the first month, I was so happy…. Because it really works. Oh my god. It made me lose weight. You know, that was perfect.’ (interview with female, 61 y with type 2 diabetes) [25]

For some patients, the weight loss gave them more confidence, which generated a positive response from their surroundings resulting in an improved social life:

‘If you’re good to yourself, you’re going to be good to everybody else and you’re going to be happier and everybody’s going to want to be around you…My social life picked up quite a bit… I started doing more and at work especially I got out of my shell. I’ve got a lot of friends now because I’m confident.’ (interview with man, 52 y with type 2 diabetes) [25]

#### 3. Patients express diverse opinions and skills regarding the medication’s usability

Two of the included studies evaluated the patients’ perspectives on the usability of injectable GLP1-RAs through qualitative semi-structured interviews. One study was based on interviews, where patients with type II diabetes had to imagine themselves taking either daily oral semaglutide, once-a-week injectable semaglutide, or once-a-week injectable dulaglutide for 20 years. The patients’ opinions in the studies showed great heterogeneity, with some being thrilled about the injectable treatment and some disliking it.

For some, injecting themselves seemed impossible, and they wanted their general practitioner (GP) to do it for them.

‘I could never inject myself. I would have to get a GP to do it for me. No way I’d inject myself.’ (patient with type 2 diabetes, gender and age not given) [23]

Even though some patients disliked the idea of injecting themselves, the prospect of weight loss motivated them to continue the treatment:

‘I didn’t really like the injection side of things and having to do it myself and all that but put that aside… [when] she (the nurse) explained to me that [GLP-1RA] would help me lose weight. I’m like, right, that’s enough motivation.’ (interview with man, 66y with type 2 diabetes) [24]

The semaglutide injection pen was also compared to the dulaglutide injection pen. Patients found the dulaglutide injection pen simpler, easier to remember, and more comfortable as the needle is not visible, when using the injection pen.

‘[The dulaglutide injection] is the easiest. Dosage is fixed, you don’t see the needle, you only do it once a week, so you don’t have to worry about it.’ (patient with type 2 diabetes, gender and age not given) [23]

Furthermore, some patients also found it easier only to inject themselves once a week than taking a daily tablet of semaglutide with restrictions:

‘You have to wait 30 minutes to eat, and you have to take it on an empty stomach. I like to have coffee first thing when I wake up. I would mess it up every day.’ (patient with type 2 diabetes, gender and age not given) [23]

The eating restrictions that followed with oral semaglutide could even affect some patients’ quality of life:

‘I don’t like the idea of having to wait to eat [referring to oral semaglutide]. It’s just going to start affecting your quality of life to have the eating restrictions.’ (patient with type 2 diabetes, gender and age not given) [23]

#### 4. Patients believe that the medication improves their ability to manage sweet cravings

Four of the included studies [4,25,26,28] assessed patients’ experiences with better management of sweet cravings through patient-reported outcomes, semi-structured interviews and questionnaires. The relationship between food cravings and restriction is complex to measure and can be affected by multiple factors (mood, physical activity, situational settings, food exposures and dysregulated eating behaviors) [26].

The survey study investigating the short-term effects of semaglutide on behavioral eating and other abnormal eating patterns found a reduction in participants’ cravings for sweets [28]. Likewise, this reduction was also illustrated in the STEP 5 study and in study that explored the wider benefits of participation in the STEP trials [4,26]. Better self-reported management of sweet cravings was also mentioned in one qualitative study:

‘I didn’t have nearly as much of an appetite, and I was not craving sugars like I had been so that certainly made it simpler to stay lower on my weight and to continue to lose weight’ (interview with man, 65 y with type 2 diabetes) [25]

#### 5. Gender seems to affect patients’ experiences with the medication, with females reporting more benefits than males

Several of the included studies illuminated a tendency for females to experience more benefits from treatment with semaglutide than males [23,26,27]. Among individuals with obesity, females reported lower health-related quality of life than men before use of the medication, particularly experiencing more pronounced negative effects of obesity on their mental health. Throughout the STEP studies, females in the intervention group (treated with semaglutide) reported notably better scores in all domains in the SF-36 than males [26].

## Discussion

### Summary of main findings

The five themes illustrate different aspects of patients’ experiences with GLP1-RAs. The findings showed that although patients did experience side effects from the medication (such as nausea and vomiting), they were willing to accept these due to their motivation to and experience of losing weight. The patients experienced improved physical functioning, and some found the injection treatment superior to oral treatment due to its less frequent dosing and fewer restrictions. For others, the medication helped to better manage their cravings for sweets. Finally, in the included studies there was a tendency that females’ physical functioning and mental well-being improved more than males.

Patients’ willingness to accept severe physical consequences and high mortality risks to lose weight, as demonstrated in other studies [29,30], reflects a profound motivation to address the personal and societal burden of obesity. In one study 20% of 654 patients were willing to accept a mortality risk greater than 10% to undergo weight loss surgery, highlighting the lengths patients might go to achieve weight loss goals [29]. Similarly, a survey of 365 primary care patients living with obesity revealed that a third were willing to trade lifetime for a 10% weight loss [30]. These findings align with the theme that patients prioritize immediate weight loss outcomes, often over long-term health considerations, a theme also reported in other studies [31]. However, our review suggests that this willingness might be modulated by patients’ quality of life (QoL) scores, as patients with higher QoL scores tend to be more risk-averse [29,30].

Adding to this, our synthesis highlights the economic burden patients are willing to shoulder. For example, in a psychological and behavioral survey [31], participants were willing to pay $30 monthly for weight loss medication, a figure far exceeded by the current prices of GLP1-RAs, such as Wegovy and Ozempic [31,32]. This willingness to invest financially further underscores the perceived value of these medications in achieving weight-related goals. Nevertheless, the disparity between affordability and accessibility raises important ethical and policy implications, particularly for patients in lower socioeconomic brackets.

The high demand for GLP1-RAs has created significant pressure on primary care providers, who face the dual challenge of meeting patient expectations while balancing clinical guidelines [6,7,32]. This tension is particularly pronounced given the broader metabolic benefits of GLP1-RAs, such as improved cardiovascular risk profiles, which may further amplify patient interest [33].

However, despite this growing demand, there remains a notable absence of comprehensive qualitative studies exploring the long-term patient experience with GLP1-RAs. This gap is especially critical given the reported gender difference in perceived benefits as described in theme five. Women, who often report lower baseline Quality of Life, tend to experience greater improvements in physical and mental health outcomes compared to men, as revealed in the STEP studies [26,27].

While the findings of this review demonstrate a strong patient motivation to use GLP1-RAs despite side effects and high costs, they also call for a more holistic understanding of patient perspectives. For instance, qualitative research could explore how patients navigate the trade-offs between adverse effects, usability and financial burden in real-world settings. Similarly, there is a need to investigate the broader psychosocial impacts of GLP1-RA use, particularly regarding self-image, social relationships and adherence to lifelong treatment.

### Strengths and limitations

This review has several methodological strengths: first, it was conducted by a multidisciplinary research team (CKI is a medical doctor, MBK is a political scientist, SO is an anthropologist, AKLC holds a master’s in molecular medicine, CTS is a sociologist and holds a PhD in qualitative science communication, RR is a senior resident in endocrinology and GO is a language psychologist) several are well-rehearsed in qualitative methods. Second, the systematic review was structured with comprehensive, and iterative search strategy. The iterative search went through citations in the included studies and selected articles from PubMed, decreasing the risk of missing relevant articles. The use of the COREQ-, CONSORT- and STROBE checklists to assess the quality in reporting was also considered a strength. We consider this to reflect good quality in reporting, based on standards referenced in the literature [34].

While the primary research interest was qualitative by nature, focusing on the patient’s experiences, the value of including diverse study designs was recognized. Both quantitative and qualitative data were incorporated and analyzed through a qualitative lens, which allowed us to capture a multi-dimensional view of patient experiences. This is considered a strength in the systematic review, and the approach has also been used in prior systematic reviews [35].

The scarcity of literature was a limitation of this systematic review. Likewise, the inclusion of type II diabetes patients’ experiences with GLP1-RAs was a limitation because people with type II diabetes might have a different experience with GLP1-RAs compared with patients only living with obesity. Finally, the absence of a quality assessment tool for the two narrative reviews was noted.

### Limitations related to the included studies

A limitation was the inability to access raw data from the STEP studies, affecting the interpretation of the final data. In the studies using questionnaires patient responses were mostly limited to predetermined questions, which may not fully capture their experiences with GLP1-RAs. Qualitative interviews are suggested as more effective for exploring patient experiences, offering in-depth understanding and flexibility. Funding from Novo Nordisk and Eli Lilly in six studies may potentially influence the interpretation in these studies. Therefore, there is a need for further research that is more product independent. The STEP studies’ population was the largest, but with a demographic bias toward female and Caucasic participants, suggesting a need for broader representation [1–4,26,27].

### Implications for research

This systematic review demonstrated that there is a major gap in research on GLP1-RAs medication regarding patients’ experiences leaving great potential for future qualitative research to fill this gap and shed light on the broader aspects of the patient’s experiences with GLP1-RAs. Furthermore, future research should delve deeper into gendered nuances, as well as explore how cultural and socioeconomic factors shape patient experiences.

### Implication for clinical practice

Despite the scarcity of qualitative research, an implication for clinical practice is the knowledge that nausea is a frequent side effect during the dose escalation period with GLP1-RAs. The frequency of nausea experienced is reduced after this period, and in some patients, it disappears completely. It is also worth considering that for many patients, their motivation to lose weight outweighs the side effects. However, this systematic review indicates that patients primarily highlight their benefits of weight loss rather than the specific weight loss method used to achieve it (e.g. semaglutide). Another weight reduction method with fewer side effects could potentially yield similar improvements in patients’ physical functioning, activities of daily living and well-being.

## Conclusion

This systematic review has synthesized available literature on patients’ experiences with GLP1-RAs. Patients are willing to accept even severe physical consequences to achieve weight loss. Despite a huge demand and usage, there is almost no qualitative research on the patients’ perspectives and experiences. Therefore, there is a need for studies to investigate the patient’s experiences with GLP1-RAs in both the short and long term.

## Supplementary Material

Supplementary file 2.docx

Supplementary file 1.docx

## Data Availability

All data in this review are based on an analysis of published papers. The appendix includes the search string.
