# Supplementary material for: Patients’ experiences with GLP1-RAs – a systematic review
Source: Scand J Prim Health Care. 2025 Mar 12;43(2):370–9. doi: 10.1080/02813432.2025.2477141 (PMC12090293; doi:10.1080/02813432.2025.2477141)
Supplement: Supplementary file 2.docx [file IPRI_A_2477141_SM2149.docx]

# **Supplementary file 2**

## **1. Search string**

**Search string in PubMed and EMBASE:**

- semaglutide
- Wegovy
- Ozempic
- Rybelsus
- tirzepatide
- Mounjaro
- lixisenatide
- Lyxumia
- (semaglutide) AND (experience)
- (semaglutide) AND (perception)
- (semaglutide) AND (perspective)
- (semaglutide) AND (preference)
- (semaglutide) AND (attitude)
- (dulaglutide) AND (experience)
- (dulaglutide) AND (perception)
- (dulaglutide) AND (perspective)
- (dulaglutide) AND (preference)
- (dulaglutide) AND (attitude)
- (liraglutide) AND (experience)
- (liraglutide) AND (perception)
- (liraglutide) AND (perspective)
- (liraglutide) AND (preference)
- (liraglutide) AND (attitude)
- (exenatide) AND (experience)
- (exenatide) AND (perception)
- (exenatide) AND (perspective)
- (exenatide) AND (preference)
- (exenatide) AND (attitude)
- (Ozempic) AND (experience)
- (Ozempic) AND (perception)
- (Ozempic) AND (perspective)
- (Ozempic) AND (preference)
- (Ozempic) AND (attitude)
- (Wegovy) AND (experience)
- (Wegovy) AND (perception)
- (Wegovy) AND (perspective)
- (Wegovy) AND (preference)
- (Wegovy) AND (attitude)
- (Rybelsus) AND (experience)
- (Rybelsus) AND (perception)
- (Rybelsus) AND (perspective)
- (Rybelsus) AND (preference)
- (Rybelsus) AND (attitude)
- (Saxenda) AND (experience)
- (Saxenda) AND (perception)
- (Saxenda) AND (perspective)
- (Saxenda) AND (preference)
- (Saxenda) AND (attitude)
- (Trulicity) AND (experience)
- (Trulicity) AND (perception)
- (Trulicity) AND (perspective)
- (Trulicity) AND (preference)
- (Trulicity) AND (attitude)
- (Byetta) AND (experience)
- (Byetta) AND (perception)
- (Byetta) AND (perspective)
- (Byetta) AND (preference)
- (Byetta) AND (attitude)
- (Bydureon) AND (experience)
- (Bydureon) AND (perception)
- (Bydureon) AND (perspective)
- (Bydureon) AND (preference)
- (Bydureon) AND (attitude)
- (Glucagon-like-Peptide-1 receptor agonist)) AND (experience)
- (Glucagon-like-Peptide-1 receptor agonist)) AND (perception)
- (Glucagon-like-Peptide-1 receptor agonist)) AND (perspective)
- (Glucagon-like-Peptide-1 receptor agonist)) AND (preference)
- (Glucagon-like-Peptide-1 receptor agonist)) AND (attitude)

**Search string in PsycINFO and Sociological abstract:**

- semaglutide
- dulaglutide
- tirzepatide
- liraglutid
- lixisenatide
- exenatid
- Saxenda
- Ozempic
- Wegovy
- Rybelsus
- Lyxumia
- Mounjaro
- Trulicity
- Byetta
- Bydureon
- (Glucagon-like-Peptide-1 receptor agonist)) AND (experience)
- (Glucagon-like-Peptide-1 receptor agonist)) AND (perception)
- (Glucagon-like-Peptide-1 receptor agonist)) AND (perspective)
- (Glucagon-like-Peptide-1 receptor agonist)) AND (preference)
- (Glucagon-like-Peptide-1 receptor agonist)) AND (attitude)
- (Ozempic) AND (experience)
- (Ozempic) AND (perception)
- (Ozempic) AND (perspective)
- (Ozempic) AND (preference)
- (Ozempic) AND (attitude)
- (Wegovy) AND (experience)
- (Wegovy) AND (perception)
- (Wegovy) AND (perspective)
- (Wegovy) AND (preference)
- (Wegovy) AND (attitude)
- (semaglutide) AND (experience)
- (semaglutide) AND (perception)
- (semaglutide) AND (perspective)
- (semaglutide) AND (preference)
- (semaglutide) AND (attitude)

**Mesh terms used in Pubmed:**

("Glucagon-Like Peptide 1"[Mesh]) AND ([experience* OR attitude OR perception OR perspective OR preference])
