# Supplementary material for: Patients’ experiences with GLP1-RAs – a systematic review
Source: Scand J Prim Health Care. 2025 Mar 12;43(2):370–9. doi: 10.1080/02813432.2025.2477141 (PMC12090293; doi:10.1080/02813432.2025.2477141)
Supplement: Supplementary file 1.docx [file IPRI_A_2477141_SM2148.docx]

# Supplementary file 1


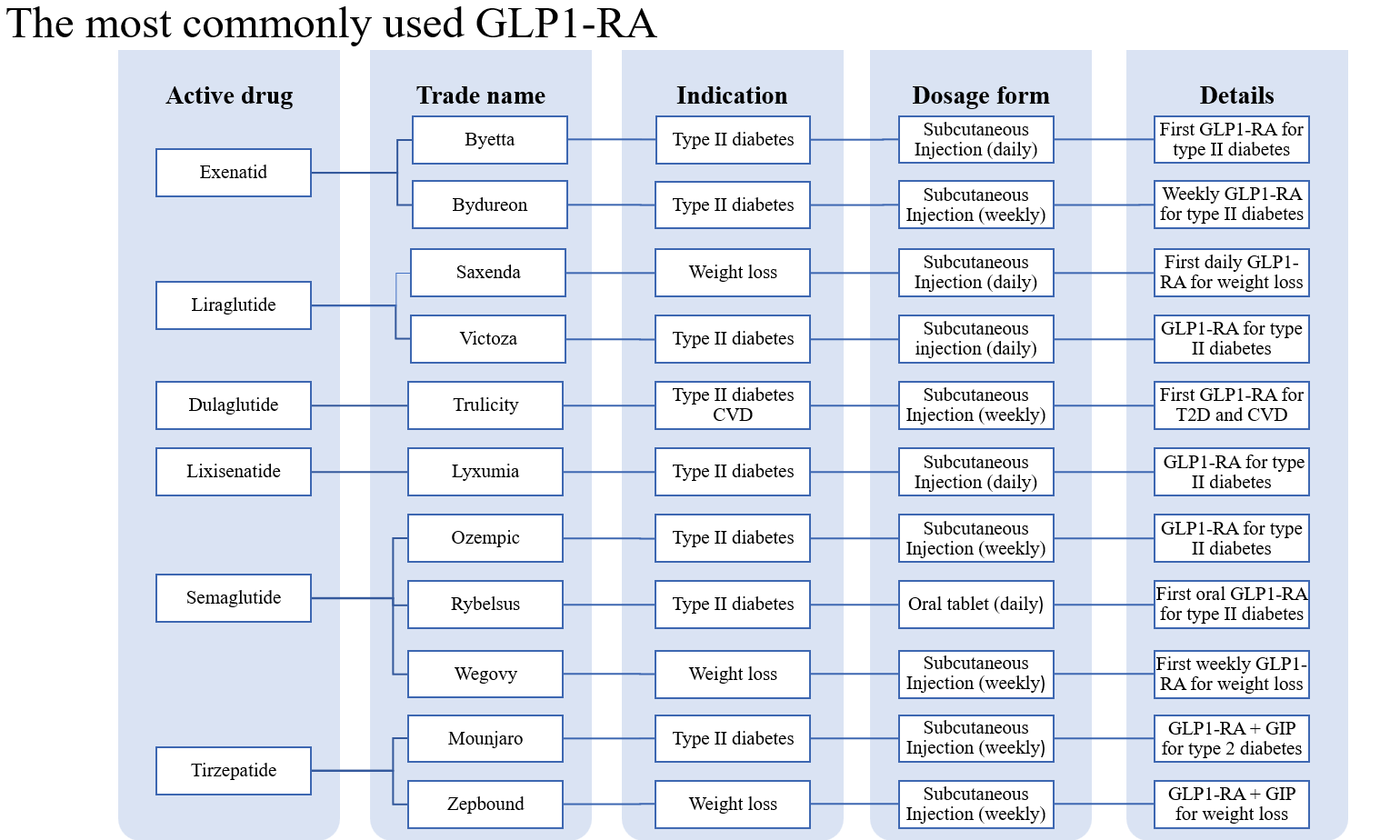


Figure 2: “The most commonly used GLP1-RA” with relevant references[1, 2]

1. Collins, L. and R.A. Costello, *Glucagon-like peptide-1 receptor agonists.* 2019.

2. Danielsen, M.K. *Wegovy™ (semaglutide 2.4 mg), the first and only once-weekly GLP-1 therapy for weight management, approved in the US*. 2021.
